# A fuzzy ontology-based case-based reasoning system for stomach dystemperament in Persian medicine

**DOI:** 10.1371/journal.pone.0309722

**Published:** 2024-10-24

**Authors:** Hassan Shojaee-Mend, Haleh Ayatollahi, Azam Abdolahadi

**Affiliations:** 1 Infectious Diseases Research Center, Gonabad University of Medical Sciences, Gonabad, Iran; 2 Health Management and Economics Research Center, Health Management Research Institute, Iran University of Medical Sciences, Tehran, Iran; 3 Department of Complementary Medicine, Research Institute for Islamic and Complementary Medicine, Iran University of Medical Sciences, Tehran, Iran; West Pomeranian University of Technology, POLAND

## Abstract

**Background:**

In Persian medicine, early diagnosis and treatment of stomach dystemperament is crucial for preventing other diseases. However, traditional medicine diagnosis often involves ambiguous and less structured information making it challenging for practitioners. Integrating fuzzy ontology with case-based reasoning (CBR) systems can enhance diagnostic accuracy in this filed.

**Objectives:**

This study aimed to develop and evaluate a fuzzy ontology-based CBR system for diagnosing and treating stomach dystemperament in Persian medicine.

**Methods:**

This was a mixed-methods research in which a fuzzy ontology-based CBR system was developed based on the fuzzy features, utilizing trapezoidal, triangular, right shoulder and left shoulder membership functions to represent linguistic variables such as hunger level and digestion power. The research phases included identifying relevant terms, concepts, and relationships, developing the fuzzy case-base ontology using the IKARUS-Onto methodology, and subsequently designing and implementing the CBR system. The system performance was evaluated in terms of its sensitivity, specificity, accuracy, precision, and F1-score.

**Results:**

Initially, a case-base fuzzy ontology was created. Then, the database was built up using 88 expert-validated medical records. Of these cases, 72% (63 cases) were diagnosed with phlegmatic dystemperament, 18% (16 cases) with cold-dry dystemperament, and 10% (9 cases) had no stomach dystemperament. The CBR system was developed and evaluated using sensitivity, specificity, accuracy, precision, and F1-score which were 97.5%, 87.5%, 96.6%, 98.7%, and 98.1%, respectively.

**Conclusions:**

Our fuzzy ontology-based CBR demonstrated high performance in diagnosing stomach dystemperament in Persian medicine. This system shows promise in improving diagnostic accuracy and facilitating the identification of similar cases. While initial results are encouraging, further evaluation in a real clinical environment is recommended to fully assess its practical utility.

## Introduction

Traditional medicine encompasses practices rooted in indigenous cultures worldwide, and has long been used to maintain health and treat illnesses [1]. The World Health Organization (WHO) accepted this specialty and emphasized its accessibility and cost-effectiveness [1, 2].

Persian medicine with a history spanning over 1000 years, is one of the oldest types of traditional medicine [3]. In Persian medicine, the digestive system, particularly the stomach, holds great significance due to its perceived connection with other bodily organs. Many diseases are believed to be related to digestive system disturbances, especially improper stomach function [4, 5]. Consequently, early diagnosis and treatment of stomach dystemperament is considered crucial for preventing various diseases [6].

However, traditional medicine, including Persian medicine, faces several challenges. Diagnosis often relies heavily on the physician’s experience, and information is not always well-documented or standardized [7, 8]. Moreover, in traditional medicine, clinical data often relies on qualitative descriptions and subjective symptoms rather than quantitative measurements. This is particularly evident in diagnosing stomach dystemperament, where most indicators are subjective and expressed in natural language. [5] These factors make collecting domain-specific data challenging and highlight the potential value of experience in supporting clinical decisions [9].

Case-based reasoning (CBR) is a technology that has shown promises in various medical fields, but has received less attention in traditional medicine [10–12]. CBR systems can enhance decision-making quality and reduce medical errors [8]. However, designing such systems for traditional medicine is challenging due to the unstructured nature of clinical information and the lack of well-described specialized knowledge [13]. To address these challenges, the development and use of medical ontology as the foundation for CBR systems can be highly beneficial [14]. Medical ontology provides background knowledge and facilitates semantic retrieval, which becomes increasingly important as the number of cases grows [15]. Furthermore, fuzzy ontology can accommodate the ambiguous knowledge often present in traditional medicine [16].

While several CBR systems have been introduced in traditional Chinese medicine [17, 18], limited research has been conducted on Persian medicine ontology and CBR system development [19, 20]. Given that Persian medicine knowledge is largely experience-based and involves ambiguity, developing clinical decision support systems such as CBRs, supported by fuzzy ontology, could enhance the efficiency of Persian medicine specialists in diagnosing diseases. This study aimed to address the following research question: How can we develop a fuzzy ontology-based CBR system to support the diagnosis and treatment of stomach dystemperament in Persian medicine? The main objectives of this research were to develop a fuzzy ontology for stomach dystemperament in Persian medicine, design and implement a CBR system based on the fuzzy ontology, and evaluate the effectiveness of the proposed system in supporting clinical decision-making for stomach dystemperament diagnosis and treatment.

## Materials and methods

This study, conducted in 2021, used anonymized data extracted from the medical records of patients referred to a traditional medical center. The patients’ medical records were accessed between April 18 and May 6, 2021. Before conducting the research, ethics approval was sought from the ethics committee of Iran University of Medical Sciences (IR.IUMS.REC.1397.440).

A data collection form was used to extract relevant information from the medical paper records of 100 patients. Initially, a case base for the CBR system was created using the available data in the medical records of 100 patients. The data for each patient consisted of personal information, physical examination results, gastrointestinal symptoms, temperament attributes, pulse characteristics, thirst levels, appetite, stool status, associated diseases, and diagnoses. Upon initial examination, we found that most of the records were incomplete with large amounts of missing data. To ensure the quality and reliability of the case base, listwise deletion method was used. Records with more than 25% missing data (12 fields) were removed from the dataset. This resulted in a final case base of 88 records, each containing 52 features.

### Methodological approach

**Fig 1:**
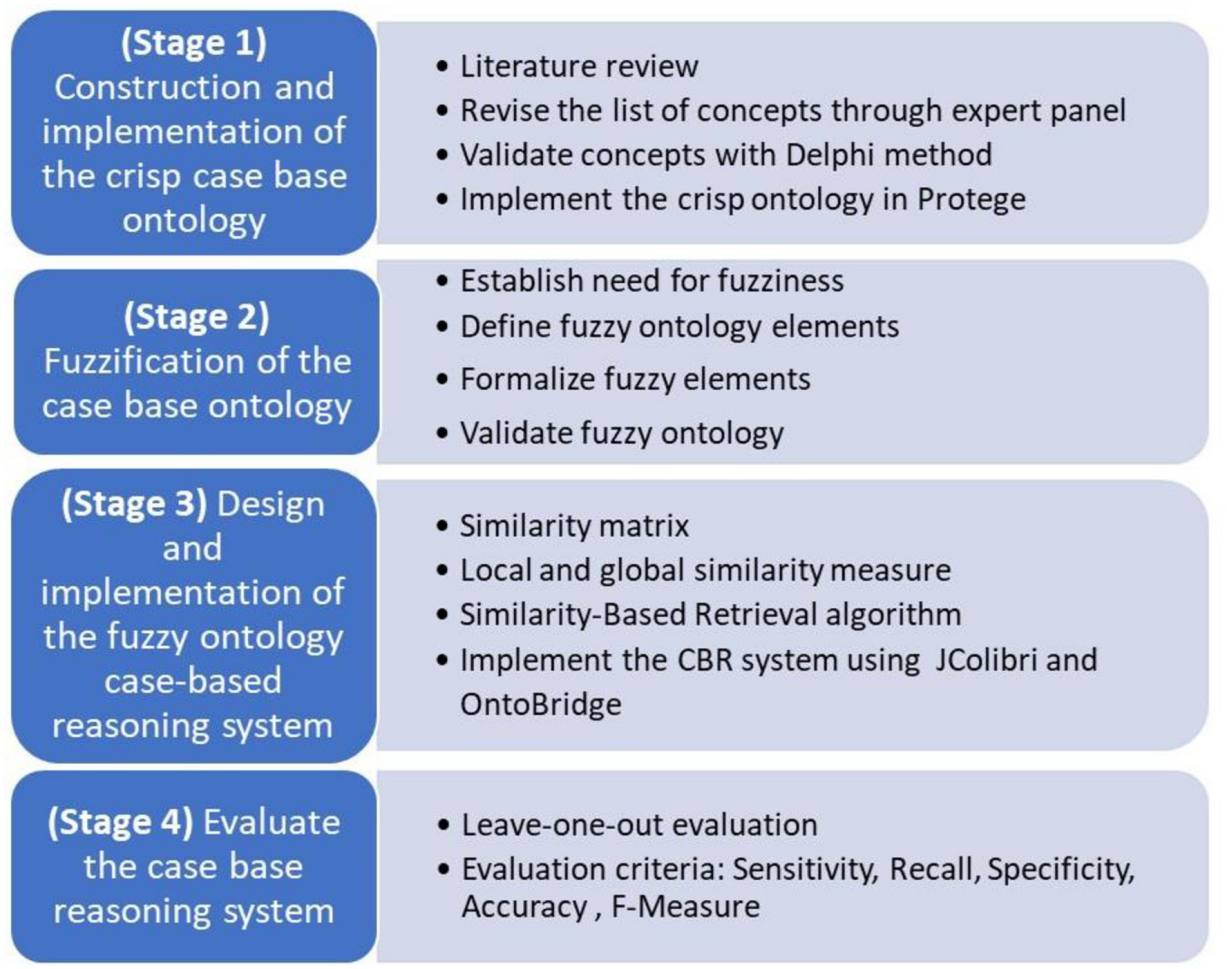
Stages for developing the fuzzy ontology CBR system.

A fuzzy ontology-based CBR system was developed in four stages for the diagnosis and treatment of stomach dystemperament in Persian medicine (Fig 1).

#### Stage 1: Construction of the crisp case base ontology

Both qualitative and quantitative approaches were used to construct the crisp case base ontology consisting of three steps. First, the concepts and relationships related to stomach dystemperament in Persian medicine were identified through reading the textbooks in Persian medicine and relevant articles. In the second step, the opinions of the Persian medicine experts regarding the identified concepts were collected through expert panel discussions. Subsequently, concept validation was performed using the Delphi technique, and the crisp ontology was created and evaluated using the approved concepts [21].

#### Stage 2: Fuzzification of the case base ontology

The fuzzification process was implemented to account for the inherent uncertainty in medical diagnoses, particularly in Persian medicine. The IKARUS-Onto approach was used to develop the fuzzy ontology, which is applicable when a crisp ontology of the domain of interest exists [22]. In this study, the crisp ontology created in the first stage served as a starting point for developing the fuzzy ontology. The fuzzification process involved identifying the fuzzy concepts that required fuzzy representation based on their inherent vagueness or variability in Persian medicine. Working with Persian medicine experts, we defined appropriate fuzzy sets and membership functions for each identified fuzzy concept. Then, we used the FuzzyOWL2 plugin in Protege software to implement the fuzzy concepts within the ontology. Finally, fuzzy ontology was evaluated using four criteria: accuracy, completeness, and consistency. The first two criteria were assessed through the expert evaluation, while consistency was checked using fuzzy ontology reasoners [22].

#### Stage 3: Designing and implementing a fuzzy ontology CBR system

The CBR system was designed to utilize the fuzzy case-base ontology. For fuzzy features, membership degrees were calculated and incorporated into the fuzzy ontology. We used a two-stage similarity algorithm for case comparison. The local similarity calculated feature-level similarity using different methods based on the data types (numerical, nominal, textual, fuzzy, ordinal, and ontological). The global similarity computed overall case similarity using a weighted average of local similarities. To address the missing data issues, we employed a combination of listwise deletion method for cases with more than 25% missing values, and pairwise deletion during case comparison. Only features that had values for both cases were considered in the comparison. The final system was designed using Java, Eclipse IDE, and the JCOLIBRI3 and OntoBridge frameworks.

*Calculation of local similarity*. Features were divided into six types based on the nature of information they contained: numerical, nominal, textual, ordinal, fuzzy, and ontological. Each feature type required a specific method for calculating local similarity.

The local similarity for numerical features was calculated based on Eq 1. In this equation, q_i_ and c_i_ represent the values associated with numerical feature i in two instances, q and c. max_i_ and min_i_ are the maximum and minimum values of feature i in the dataset, respectively.


$$sim_{Num}\left(q_{i},c_{i}\right)=1-\frac{\left|q_{i}-c_{i}\right|}{max_{i}-min_{i}}$$ (1)

Eq 2 was used to calculate local similarity for nominal values. In this equation, q_i_ and c_i_ are the values associated with nominal feature i in instances q and c, respectively.


$$sim_{Nom}\left(q_{i},c_{i}\right)=\left\{\begin{array}{ll} 1 & q_{i}=c_{i}\\ 0 & q_{i}\neq c_{i} \end{array}\right.$$ (2)

Some features in Persian Medicine contained textual expressions. To calculate local similarity for these textual features, the Jaccard similarity coefficient [23], as defined in Eq 3, was used. In this equation, the values of two features, q_i_ and c_i_, are represented as sets of textual expressions.


$$sim_{Txt}\left(q_{i},c_{i}\right)=\frac{\left|q_{i}\cap c_{i}\right|}{\left|q_{i}\cup c_{i}\right|}$$ (3)

For ordinal features, the recommended similarity matrix proposed by the experts in Persian medicine was used to calculate local similarity. The first part of the equation *sim*_*Ord*_(*q*_*i*_, *c*_*i*_) determines the degree of similarity between two ordinal values, q_i_ and c_i_, using similarity matrices.

For fuzzy features, similarity between two fuzzy linguistic terms was calculated using a method similar to El-Sappagh et al. [14], and involved similarity matrices proposed by experts in Persian medicine. The local similarity between two fuzzy feature values was calculated through Eq 4. In this equation, q_i_ and c_i_ are two numerical values related to fuzzy feature i, and n is the number of membership functions defined for the fuzzy feature. μ_k_ represents the membership function k of the fuzzy feature.


$$sim_{Fzy}\left(q_{i}.c_{i}\right)=1-\sum_{k=1}^{n}\frac{\left|\mu_{k}\left(q_{i}\right)-\mu_{k}\left(c_{i}\right)\right|}{n}$$ (4)

Ontological features, specifically the “associated diseases” feature, required a semantic similarity calculation. We implemented a hybrid approach combining the Wu-Palmer and Lin methods (Eq 5). The Wu-Palmer method considers the depth of concepts in the ontology hierarchy, while the Lin method incorporates information content. This combination allows for a comparison of diseases based on both their hierarchical relationships and their specificity within the ontology.


$$Sim_{Sem}\left(C1.C2\right)=\;\frac{Sim_{Wu\&Palmer}\left(C1.C2\right)+Sim_{Lin}\left(C1.C2\right)}{2}$$ (5)

*Calculation of global similarity*. Since ontology-based modeling was used for cases, the similarity between two cases can be calculated based on the similarity of their objects and data properties [24]. For this purpose, Eq 6 was used.


$$Sim_{I}\left(C1.C2\right)=\frac{\sum_{i=1}^{m}\left(W_{i}\times Sim_{a}\left(a_{i}^{C1}.a_{i}^{C2}\right)\right)+\sum_{j=1}^{n}\left(W_{j}\times Sim_{I}\left(Ir_{j}^{C1}.Ir_{j}^{C2}\right)\right)}{\sum_{i=1}^{m}\;W_{i}+\sum_{j=1}^{n}\;W_{j}}$$ (6)

This method computes a weighted average of local similarities for all features, with weights determined by Persian medicine experts to reflect the relative importance of each feature in diagnosis. In the above equation, m represents the number of data properties, and n represents the number of object properties in samples C1 and C2. a_i_^C1^ refers to the value of the i-th data property related to C1, and a_i_^C2^ is the value of the i-th data property related to C2. Sim_a_ calculates the similarity between two features based on the equations defined in the previous section for local similarity calculation. Ir_j_^C1^ represents a sample related to the j-th object property of C1, and Ir_j_^C2^ is a sample related to the j-th object property of C2.

#### Stage 4: CBR system evaluation

To evaluate the system performance, the leave-one-out validation method was used. In this method, one of the cases was sequentially omitted, and the diagnosis of stomach dystemperament for that case was made using other cases in the case base. This approach was chosen, because it maximizes the use of available data for both training and testing and provides a robust estimate of the system performance on the unseen cases. Performance metrics including specificity, sensitivity (recall), accuracy, precision, and F1-score were calculated to provide a comprehensive evaluation of the system performance.

## Results

The results of each research stage were as follows:

### Stage 1: Construction of the crisp case-base ontology

To determine the concepts related to the diagnosis and treatment of stomach dystemperament in Persian medicine, an extensive review of relevant textbooks and articles conducted. To validate and refine our findings, the opinions of five Persian medicine specialists were collected through expert panel discussions. To further enhance the robustness of the ontology, the Delphi method was used to gather opinions from more experts to finalize the desired concepts and relationships. Subsequently, using the identified concepts and relationships, the CBRDystempOnto ontology was developed as the initial ontology for diagnosing and treating stomach dystemperament in Persian medicine using Protege software [21].

### Stage 2: Fuzzification of the case base ontology

**Fig 2:**
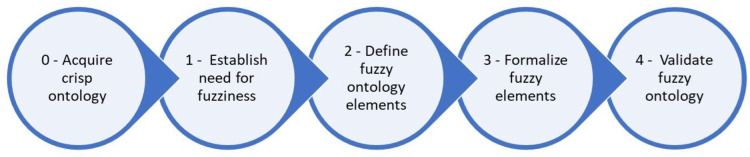
The IKARUS-Onto methodology for the development of fuzzy ontology [22].

In the second stage of our research, the IKARUS-Onto methodology was used for fuzzifying the crisp case-base ontology developed in stage 1. This methodology is applicable when a crisp ontology of the target domain already exists [22]. This approach allowed us to transform our initial ontology into a fuzzy ontology capable of handling the inherent vagueness in Persian medicine diagnostics. The IKARUS-Onto methodology consists of four key steps (Fig 2).

### 1- Establish the need for fuzziness

Domain experts confirmed that attributes such as hunger level, appetite, age, pulse rate, and digestion power contain vagueness that cannot be adequately represented in a crisp ontology [25].

### 2- Define fuzzy ontology elements

**Fig 3:**
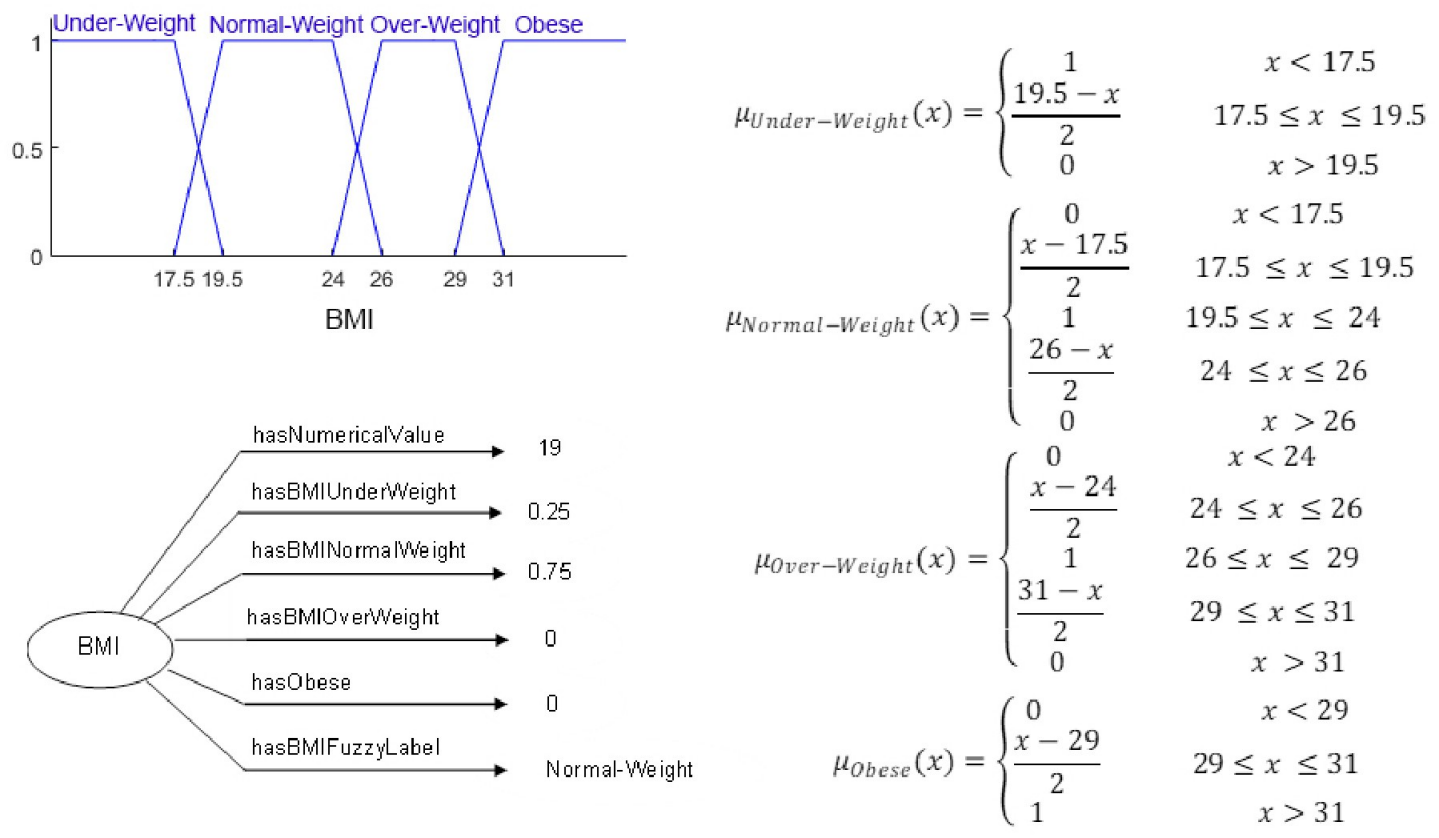
Fuzzy values and data properties for the BMI feature.

Similar to the method chosen by El-Sappagh et al. [14], the definition of fuzzy data types and modifiers was generated. For each numerical feature, a set of fuzzy values and corresponding fuzzy relations were created. As an example, for the body mass index (BMI) feature, we defined fuzzy values included underweight, normal weight, overweight, and obese, each with its own data properties to maintain numerical values, membership degrees, and fuzzy value. Fuzzy data types and data properties for the BMI feature are shown in Fig 3.

### 3- Formalize fuzzy elements

Semantic Web language version 2 (OWL 2) was used for encoding the fuzzy ontology. The ontology was implemented using Protégé version 3.4 and the FuzzyOWL plugin. For each fuzzy value, a fuzzy data type was created in Protégé. For example, the fuzzy value "Normal weight" was represented using a trapezoidal fuzzy data type.

For each numerical feature, the following fuzzy data types and data attributes were created:

- One fuzzy data type for each linguistic term.
- One data property for each linguistic term.
- One data property for the fuzzy value of the feature.
- One data property for the numerical value of the feature.

**Fig 4:**
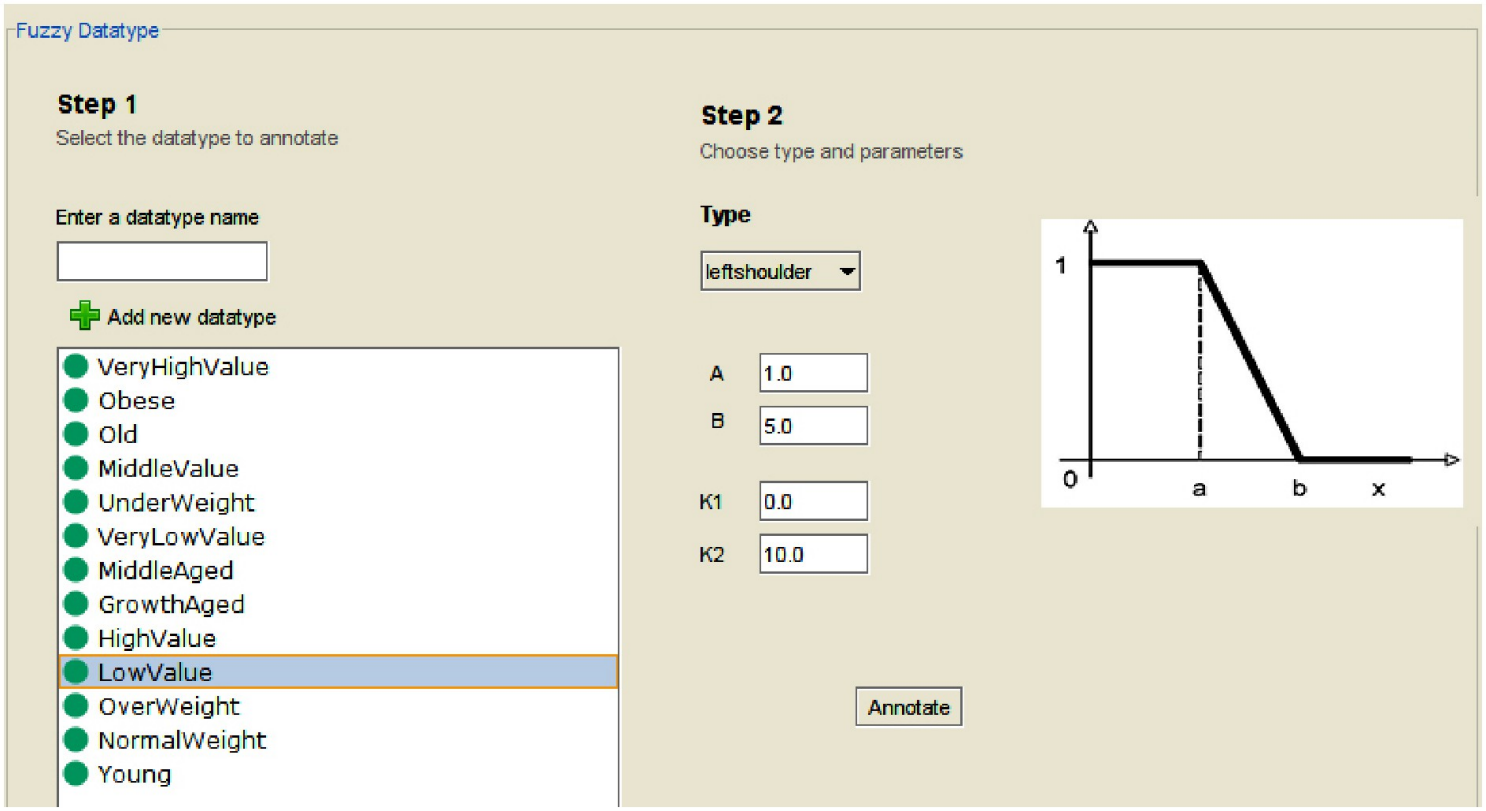
Implementation of LowValue fuzzy data type with the FuzzyOWL Plugin.

For example, the "Hunger level" feature had values in the range [0, 10], with linguistic terms such as low hunger, moderate hunger, and high hunger. For each linguistic term, a fuzzy data type was created, including Low Value, Middle Value, and High Value. Fig 4 illustrates the implementation of the left shoulder fuzzy data type for LowValue using the FuzzyOWL Plugin. In this representation, parameters K1 and K2 define the domain of the fuzzy set, where K1 is the minimum possible value and K2 is the maximum. Parameters A and B determine the shape of the left shoulder function. Specifically, A represents the highest value with full membership, while B indicates the point where membership reaches zero.

In this study, numerical features, including age, BMI, digestion power, gastric emptying rate, hunger level, appetite, pulse rate, pulse power, pulse frequency, and pulse strength, were considered fuzzy concepts. The fuzzy representation of these features, including their linguistic terms, fuzzy data types, sets, and membership degrees, is presented in Table 1.

**Table 1:** Fuzzy values, data types, sets, and membership degrees for stomach dystemperament features.

Feature	Fuzzy values	Fuzzy data type	Membership degrees
Age	Growth Aged Young, Middle Aged Old	Left Shoulder Trapezoidal Trapezoidal Right Shoulder	GrowthAged: [0, 25, 30, 150] Young:[0,25,30,40,45,150] Middle Aged:[0,40,45,60,65,150] Old: [0,60,65,150]
BMI	Underweight Normal Weight Overweight Obese	Left Shoulder Trapezoidal Trapezoidal Right Shoulder	UnderWeight: [0, 17.5, 19.5, 50] NormalWeight:[0, 17.5, 19.5, 24, 26, 50] OverWeight: [0, 24, 26, 29, 31, 50] Obese: [0, 29, 31, 50]
Digestion power Gastric emptying rate Hunger level Appetite Pulse rate pulse power Pulse frequency, Pulse strength	Low, Moderate High	Left Shoulder, Triangular, Right Shoulder	Low: [0, 1, 5, 10] Moderate: [0, 1, 5, 9, 10] High: [0, 5, 9, 10]

### 4- Validate fuzzy ontology

The fuzzy ontology was validated to ensure that domain vagueness was adequately and correctly represented. We assessed correctness by confirming with Persian medicine specialists that all selected features exhibited the vagueness. Accuracy was ensured through expert consultation on membership functions and their ranges. Completeness was verified by discussing all case-base features with Persian medicine specialists, and consistency was evaluated by converting the ontology to OWL2 format. The resulting fuzzy ontology included 79 classes, 55 object properties, 61 data properties, and 13 fuzzy data types.

### Stage 3: Design and implementation of the fuzzy ontology CBR system

**Fig 5:**
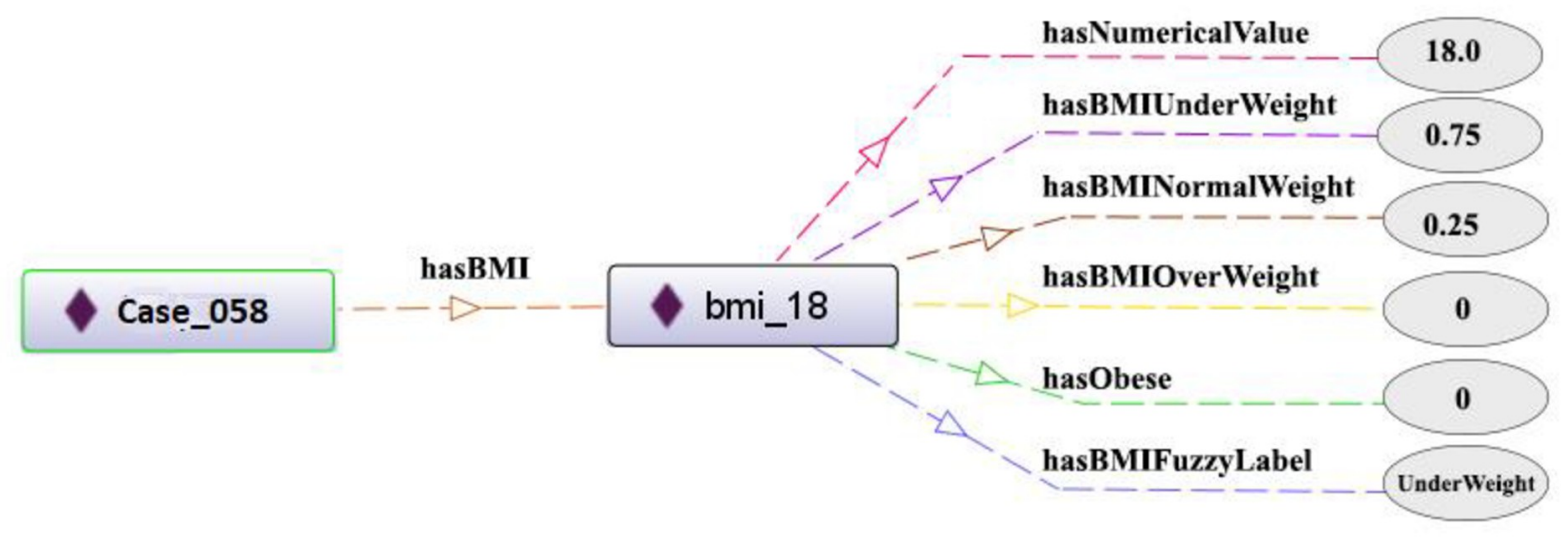
Fuzzification for an instance of BMI.

After creating instances in the ontology, the fuzzification process for fuzzy features was carried out. For instance, for the fuzzy feature BMI, four linguistic terms were considered: underweight, normal weight, overweight, and obese. As shown in Fig 5, an instance of BMI with the title "bmi_18" was created for "Case_058" in the ontology. For this instance, using the fuzzy membership functions previously created in the fuzzy ontology, membership degree values were calculated, and the necessary relations in the ontology were established. This fuzzification process was performed for all instances related to fuzzy features.

Our CBR system calculates similarities between cases in two steps: local similarity between feature values and global similarity between two cases. In this research, similarity matrices were used to calculate the local similarity between ordinal and fuzzy features. Table 2 shows the assigned values for ordinal features, and Table 3 shows the assigned values for the fuzzy feature BMI in the form of a similarity matrix.

**Table 2:** Similarity matrix for ordinal features.

Feature’s value	Very low	Low	Moderate	High	Very high
Very low	1	0.5	0	0	0
Low	0.5	1	0.7	0.5	0.2
Moderate	0	0.7	1	0.7	0.5
High	0	0.5	0.7	1	0.7
Very high	0	0.2	0.5	0.7	1

**Table 3:** Similarity matrix for linguistic terms of BMI.

Feature’s value	Under weight	Normal	Over weight	Obese
Under weight	1	0.7	0.3	0
Normal	0.7	1	0.6	0.3
Over weight	0.3	0.6	1	0.7
Obese	0	0.3	0.7	1

**Fig 6:**
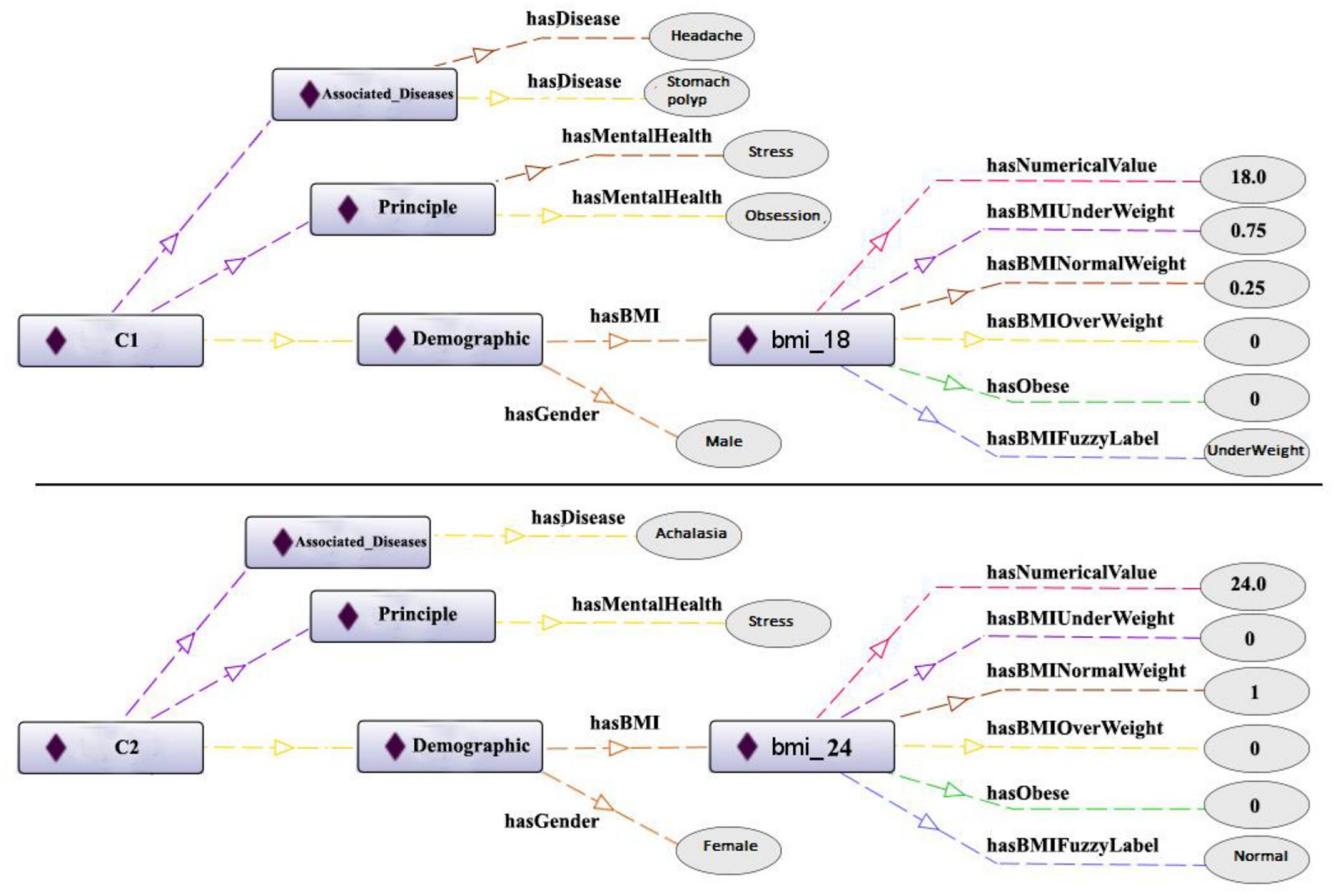
Feature values for C1 and C2 (characteristics of two patients).

The retrieval algorithm using local and global similarity methods, as illustrated in Fig 6 and based on the characteristics of two patients, is described as follows. The system considers various feature types, including BMI as a fuzzy type (Eq 7), sex as a nominal type (Eq 8), psychological symptoms (Eq 9) as a textual type, and associated diseases as ontological type (Eq 10).


$$\begin{array}{ll} sim_{Fzy-BMI}\left(18.24\right) & =1-\sum_{k=1}^{4}\frac{\left|\mu_{k}\left(18\right)-\mu_{k}\left(24\right)\right|}{4}\\ & =1-\frac{\left|\left(0.75-0\right)+\left(0.25-1\right)+\left(0-0\right)+\left(0-0\right)\right|}{4}=0.625 \end{array}$$ (7)

$$sim_{Nom}\left(\text{Male},\;Female\right)=0$$ (8)

$$sim_{Txt}\left(\left\{Stress\right\}.\left\{\text{Stress},\;\text{Obsession}\right\}\right)=\frac{\left|q_{i}\cap c_{i}\right|}{\left|q_{i}\cup c_{i}\right|}=\frac{1}{2}=0.5$$ (9)

$$Sim_{Sem}\left(Achalasia,\;Headache\right)=\;\frac{Sim_{Wu\&Palmer}+Sim_{Lin}}{2}=\frac{\frac{0}{2+3}+\frac{0}{1+1}}{2}=0$$ (10)

In the development of CBR system, we recognized the mportance of different features in diagnosing stomach dystemperament in Persian medicine. To address this, we consulted with Persian medicine specialists to assign weights to different features. Sex and demographics were given a weight of 1, BMI was assigned a weight of 3, psychological symptoms, principals (key diagnostic factors in Persian medicine), and associated diseases were given a weight of 5. Therefore, the global similarity for C1 and C2 was calculated as 0.34. (Eq 11)

$$\begin{array}{ll} Sim_{I}\left(C1.C2\right) & =\frac{1\;\times Sim_{I}\left(Demographic1.Demographic2\right)+5\;\times Sim_{I}\left(Principle1.Principle2\right)+5\times Sim_{Diseases}\left(Diseases1.Diseases2\right)}{11}\\ & =\frac{0.47+2.5+0.8}{11}=0.34 \end{array}$$ (11)

**Fig 7:**
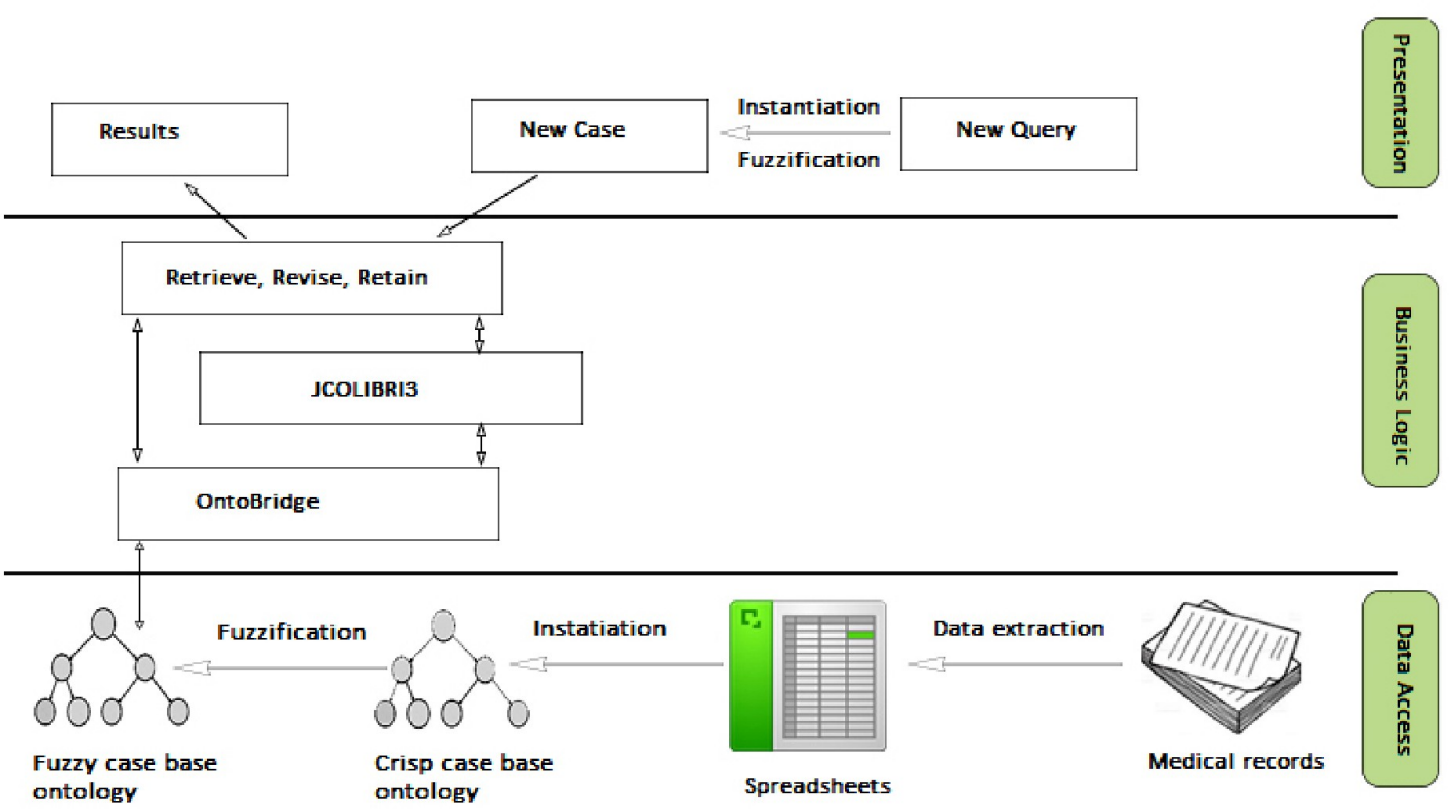
Fuzzy ontology CBR system architecture.

The system was implemented using Java along with the jCOLIBRI3 and OntoBridge frameworks. A three-tier architecture, as illustrated in Fig 7, was used for system development. The business logic layer includes modules for retrieving similar cases and revising and retaining cases in the case base. For the retrieval of similar cases, the k-nearest neighbor algorithm was used, where k = 3. The nearest neighbor algorithm used Eq 7 to calculate the global similarity between the current case and the cases stored in the case base. From this comparison, the algorithm identifies and selects the three cases with the highest similarity scores as the nearest neighbors.

Additionally, the data access layer included the fuzzy ontology of the case base, and the cases were represented as instances in the ontology. The presentation layer provides an interface for Persian medicine specialists to input queries and retrieve similar cases.

### Stage 4: CBR system evaluation

The total number of cases in the case base was 88. According to the diagnosis of Persian medicine specialists, 63 cases (72%) had phlegmatic dystemperament, 16 cases (18%) had cold-dry dystemperament, and nine cases (10%) did not have a stomach dystemperament. To evaluate the CBR system, the leave-one-out technique was used. In this method, one case was left out from the case base, and the diagnosis of the stomach temperament for that case was made by the system using the other 87 cases available in the case base. Subsequently, the system diagnosis was compared to the physician’s diagnosis. This approach was applied to all 88 cases in the case base. The results of the evaluation metrics are presented in Table 4.

**Table 4:** Evaluation metrics of the CBR system for different diagnostic classes.

Diagnostic Class	Sensitivity	Specificity	Accuracy	Precision	F1 Score
Cold-dry dystemperament	81.2	95.8	93.2	81.2	81.2
Phlegmatic dystemperament	92.2	83.3	89.8	93.6	92.9
Stomach dystemperament	97.5	87.5	96.6	98.7	98.1

As observed in Table 4, the highest accuracy (96.6%) corresponded to the diagnosis of stomach dystemperament, while the lowest accuracy (89.8%) was related to the diagnosis of phlegmatic dystemperament.

While the sample size of 88 cases may seem limited, it was appropriate for this study. Each case was carefully validated by experts, ensuring data quality. In fact, creating a large dataset in Persian medicine is challenging due to its nature and time-intensive diagnostic processes. The current dataset included various dystemperament types, allowing for comprehensive evaluation. The leave-one-out technique maximized data utilization, providing robust performance estimates. The high-performance metrics (Table 4) indicated that meaningful patterns were captured despite the sample size. Although a larger dataset could allow broader generalization, this sample offers valuable insights into the systemperformance across different dystemperament types. Future research could focus on expanding the dataset through multi-center collaborations to further validate these findings.

## Discussion

This study developed a fuzzy ontology-based CBR system for diagnosing stomach dystemperament in Persian medicine. The system achieved high accuracy in identifying stomach dystemperament (96.6%), demonstrating the effectiveness of combining fuzzy modeling with ontology reasoning to handle imprecise medical concepts. According to the literature, the use of information technology in healthcare has a significant role in reducing costs and medical errors [26]. In this study, we were able to preserve and organize knowledge related to Persian medicine by using fuzzy ontology [27].

The CBR system consists of two main parts: the case base and the retrieval algorithm [14]. Regarding the case base, one common method is to store cases in a relational database [28], which offers excellent scalability, but reasoning power will be reduced [28]. Alternatively, storing the structure of the ontology and its instances in a unified file [29] enhances ontological inference capabilities and improves the integration of ontology-oriented CBR systems. In medical applications, CBR systems have utilized diverse case base approaches. For example, Abdrabou et al. provided both file systems and relational databases, for the breast cancer CBR system [30]. In other studies, such as those for diabetes [14] and dementia care [24], the researchers used ontology-oriented approaches for their case bases. However, in the current study, both the ontology structure and instances in a unified file were adopted to maximize inference capabilities and system integration.

Having created the case base, another important part of the CBR system, is the retrieval algorithm for similar cases. In traditional medicine, this process is particularly complex due to the presence of various types of features [8]. Various methods have been used in different studies to calculate similarity based on the type of feature [8, 14, 31]. In the current research, there were five different types of features presented in the case base: numerical, nominal, ordinal, fuzzy, and ontological features. The Manhattan distance function was used for numerical features. Other functions included the equality function for nominal features, a similarity matrix for ordinal features; and a method similar to the El-Sappagh et al approach using fuzzy membership degrees for fuzzy features [14]. Additionally, there was only one ontological feature called associated diseases. A combination of Wu-Palmer’s hierarchy-based and Lin’s content-based methods was also used for measuring semantic similarity, as this combination has shown superior performance in previous studies. This comprehensive approach to similarity calculation addresses the unique challenges of case comparison in traditional medicine, contributing to the system high diagnostic accuracy.

The evaluation of the CBR system was performed using the leave-one-out technique. In the current study, the calculated values for sensitivity, specificity, accuracy, precision, and F1-score for the system were 97.5%, 87.5%, 96.6%, 98.7%, and 98.1%, respectively, indicating that the system performed well. In other similar studies, various metrics were also selected for system evaluation. For instance, Alexopoulos et al. used precision, recall, and F1-score to evaluate the performance of a CBR system based on fuzzy ontology, and they obtained values of 74%, 80%, and 77%, respectively [31].

In another study, El-Sappagh et al. applied the proposed fuzzy ontology to a CBR system for diabetes diagnosis with 60 medical records. They reported accuracy, recall, precision, feature, negative predictive value, and F1-score values of 97.67%, 96.43%, 100%, 100%, 93.75%, and 98.18%, respectively [14]. Additionally, Nasiri et al evaluated a CBR system based on ontology for dementia care using a case base with 24 cases and reported accuracy, recall, precision, and F1-score values of 60%, 60%, 83%, and 60%, respectively [24]. Therefore, it can be concluded that the selected evaluation metrics in the current research are consistent with the metrics used in other similar studies.

Despite the promising results, this study had several limitations. First of all, the sample size of 88 cases was relatively small, and may limit the generalizability of our findings. In fact, the data were collected from a single Persian medicine center, which may not represent the full spectrum of cases seen across different similar settings. However, the records were carefully examined and due the nature of Persian medicine and unstructured data, it seems that the current dataset was adequate for conducting this research. Future research should address these limitations by expanding the dataset through multi-center collaborations, conducting external validation studies, and exploring its applicability to a broader range of conditions within Persian medicine. Moreover, future research can focus on incorporating comparative analyses using different criteria and AUCROC figures.

## Conclusion

The current research successfully developed a fuzzy ontology-based CBR system for the diagnosis and treatment of stomach dystemperament in Persian medicine. The system’s case base, designed as a fuzzy ontology, enhances the quality of case representations and facilitates the search for similar cases. The CBR system showed good performance in evaluation, indicating its potential as a valuable tool for Persian medicine specialists.

The system offers several advantages that address key challenges in traditional medicine. It effectively represents similar cases and facilitates diagnosis, which is a critical aspect of Persian medicine. By providing a standardized approach to case comparison, the system has the potential to improve diagnostic consistency among practitioners. Additionally, it serves as an educational tool, helping to familiarize specialists with various cases of stomach dystemperament.

In conclusion, while the developed fuzzy ontology-based CBR system shows promise as a diagnostic tool for stomach dystemperament in Persian medicine, further research and development are needed to address its limitations and expand its capabilities. Furthermore, exploring integration with electronic health records and other clinical decision support tools could enhance its practical application in healthcare settings.
